# Outcome of Patients with Carbon Monoxide Poisoning at a Far-East Poison Center

**DOI:** 10.1371/journal.pone.0118995

**Published:** 2015-03-06

**Authors:** Chung-Hsuan Ku, Huei-Min Hung, Wa Cheong Leong, Hsiao-Hui Chen, Ja-Liang Lin, Wen-Hung Huang, Huang-Yu Yang, Cheng-Hao Weng, Che-Min Lin, Shwu-Hua Lee, I-Kuan Wang, Chih-Chia Liang, Chiz-Tzung Chang, Wey-Ran Lin, Tzung-Hai Yen

**Affiliations:** 1 Department of Nephrology and Division of Clinical Toxicology, Chang Gung Memorial Hospital and College of Medicine, Chang Gung University, Linkou, Taiwan; 2 Department of Pediatrics, Chang Gung Memorial Hospital and College of Medicine, Chang Gung University, Linkou, Taiwan; 3 Department of Psychiatry, Chang Gung Memorial Hospital and College of Medicine, Chang Gung University, Linkou, Taiwan; 4 Department of Nephrology, China Medical University Hospital and College of Medicine, China Medical University, Taichung, Taiwan; 5 Department of Gastroenterology, Chang Gung Memorial Hospital and College of Medicine, Chang Gung University, Linkou, Taiwan; 6 Kidney Research Center, Chang Gung Memorial Hospital, Linkou, Taiwan; 7 Center for Tissue Engineering, Chang Gung Memorial Hospital, Linkou, Taiwan; D'or Institute of Research and Education, BRAZIL

## Abstract

**Introduction:**

Many cases of carbon monoxide poisoning in Taiwan are due to burning charcoal. Nevertheless, few reports have analyzed the mortality rate of these patients who survive to reach a hospital and die despite intensive treatment. Therefore, this study examined the clinical features, physiological markers, and outcomes after carbon monoxide poisoning and the associations between these findings.

**Methods:**

We analyzed the records of 261 patients who were referred for management of carbon monoxide intoxication between 2000 and 2010. Patients were grouped according to status at discharge as alive (survivor, n = 242) or dead (non-survivor, n = 19). Demographic, clinical, laboratory, and mortality data were obtained for analysis.

**Results:**

Approximately half of the cases (49.4%) attempted suicide by burning charcoal. Most of the patients were middle-aged adults (33±19 years), and were referred to our hospital in a relatively short period of time (6±10 hours). Carbon monoxide produced many serious complications after exposure: fever (26.1%), hypothermia (9.6%), respiratory failure (34.1%), shock (8.4%), myocardial infarction (8.0%), gastrointestinal upset (34.9%), hepatitis (18.4%), renal failure (25.3%), coma (18.0%) and rhabdomyolysis (21.8%). Furthermore, the non-survivors suffered greater incidences of hypothermia (P<0.001), respiratory failure (P<0.001), shock (P<0.001), hepatitis ((P=0.016), renal failure (P=0.003), coma (P<0.001) than survivors. All patients were treated with high concentration of oxygen therapy using non-rebreather mask. However, hyperbaric oxygen therapy was only used in 18.8% of the patients. In a multivariate-Cox-regression model, it was revealed that shock status was a significant predictor for mortality after carbon monoxide poisoning (OR 8.696, 95% CI 2.053-37.370, P=0.003). Finally, Kaplan-Meier analysis confirmed that patients with shock suffered greater cumulative mortality than without shock (Log-rank test, Chi-square 147.404, P<0.001).

**Conclusion:**

The mortality rate for medically treated carbon monoxide-poisoned patients at our center was 7.3%. Furthermore, the analysis indicates that shock was most strongly associated with higher risk of mortality.

## Introduction

Carbon monoxide (CO) toxicity is common in Taiwan. CO is an odorless gas formed during an incomplete combustion of organic material. The most common sources of unintentional CO poisoning are faulty or inadequately ventilated gas heating appliances, fires, and automobile exhaust fumes. Indeed, between 1997 and 2003, there was a significant increase in the rate of unintentional deaths from CO poisoning in Taiwan (i.e., from 1.6 to 3.5 per 10^6^ person-years) [1].

The CO poisoning can be intentionally utilized as a form of suicide by burning charcoal and exposing themselves to the smoke. First reported in Hong Kong in 1998, suicide by charcoal burning has had an epidemic spread in Asia [2]. For example, the ratio of national suicide rate from 1999 to 2009 in Taiwan had increased from 10.4 to 19.3 for every 100,000 people [3]. During the same period, the incidence of suicide by charcoal burning alone had multiplied by nearly 25-fold, which is from 0.22 to 5.4 for every 100,000 people in Taiwan [3]. Charcoal burning soon became the second most common method of suicide and led to a 20% increase in the overall suicide rate [4]. In a preliminary study at the Chang Gung Memorial Hospital [5], it was reported that most patients that attempted suicide by charcoal burning had underlying major depressive (49.3%) or adjustment (41.1%) disorders. Breaking-up (17.8%), financial debt (17.8%), and physical/mental illnesses (17.8%) were the top three reasons for committing suicide.

The symptoms of CO poisoning are non-specific [6]. Mild exposure to CO causes headache, myalgia or dizziness whereas severe exposure will result in confusion, loss of consciousness or death [7]. Patients usually do not recognize the fact that they were exposed to CO. Every organ in human body could be damaged due to CO poisoning. However, the brain and heart with high metabolic rate are most susceptible to it. Carbon monoxide causes hypoxia by forming carboxyhemoglobin and shifting the oxyhemoglobin dissociation curve to the left [6]. The carboxyhemoglobin’s affinity for hemoglobin is more than 200 times that of oxygen [8], resulting in the formation of carboxyhemoglobin with even relatively low amounts of inhaled CO. Hypoxic brain damage predominates in the cerebral cortex, cerebral white matter, and basal ganglia, especially in the globus pallidus. Carbon monoxide poisoning also induces cellular changes, including immunological and inflammatory damage [9]. The effects of this damage are long lasting and are independent of hypoxia [9]. Since the symptoms of CO poisoning are variable and nonspecific, the only way to truly diagnose CO poisoning is serum carboxyhemoglobin levels.

Treatment of CO poisoning begins with inhalation of a high concentration oxygen and aggressive supportive care. Hyperbaric oxygen therapy accelerates the dissociation of CO from hemoglobin and may prevent delayed neurologic sequelae [10]. Nevertheless, the indications for hyperbaric oxygen therapy for CO poisoning remain controversial [10], and the ideal regimen of oxygen therapy is yet to be determined, and significant controversy exists regarding hyperbaric oxygen therapy protocols.

Few reports in Taiwan [1, 11] have evaluated the mortality rate of CO-poisoned patients that had survived upon arrival to the hospital, but died later, despite intensive medical care. Furthermore, the baseline characteristics of CO poisoning in Taiwan [5] may differ from those of other international Poison Centers, where only few of the CO poisonings may be due to charcoal burning. Therefore, the objective of this study was to examine the clinical features, physiological markers, and clinical outcomes after CO poisoning and the associations between these findings.

## Materials and Methods

### Ethics

The present retrospective observational study complied with the guidelines of the Declaration of Helsinki, and was approved by the Medical Ethics Committee of Chang Gung Memorial Hospital, a tertiary referral center (with 24-hour hyperbaric oxygen service) located in the northern part of Taiwan. Since this study involved a retrospective review of existing data, Institutional Review Board approval was obtained without specific informed consent from the patients. However, informed consent was obtained from all patients at their initial admission for risk of acute CO poisoning and all treatments. Additionally, all individual information was securely protected by delinking identifying information from main data set and was only available to investigators. Furthermore, all of the data were analyzed anonymously. The Institutional Review Board of the Chang Gung Memorial Hospital had specifically waived the need for consent. Finally, all primary data were collected according to the strengthening the reporting of observational studies in epidemiology guidelines. This policy was based on previous publications [12, 13].

### Patients

The medical records of 261 patients with acute CO poisoning that were seen at the Chang Gung Memorial Hospital between 2000 and 2010 were examined. Demographic, clinical, and laboratory data were collected, and the mortality rate was determined. Diagnosis of CO intoxication was based on clinical history, physical and laboratory examination, and was confirmed by the blood carboxyhemoglobin test.

### Inclusion and exclusion criteria

All patients older than 18 years of age that were diagnosed with CO poisoning at the Chang Gung Memorial Hospital between 2000 and 2010 were eligible for inclusion in this study. Patients were excluded from this study if they were younger than 18 years old. Additionally, patients were excluded if they did not have detectable carboxyhemoglobin levels in their blood, despite a suspicious history of exposure or the presence of major systemic comorbidities, such as cancer or heart, lung, renal, or liver diseases.

### Detoxification protocol

Treatments included administering a high concentration of oxygen therapy via a non-rebreather mask or providing hyperbaric oxygen therapy. Similar to other international Poison Centers [14], there was no standard indication for such hyperbaric oxygen treatment. Furthermore, the National Health Insurance of Taiwan did not cover the cost of hyperbaric oxygen therapy. Finally, CO patients also received full medical support and treatments for various complications.

### Definitions of clinical events

Hypothermia was defined as a body temperature of below 36.0°C [15]. Shock was defined as an abnormality of the circulatory system that results in inadequate organ perfusion and tissue oxygenation [16, 17]. Acute hepatitis was diagnosed if there were increases in alanine aminotransferase levels greater than 2 times of upper normal limit (i.e., >68 U/L, normal: 0–34 U/L) or total bilirubin levels were >1.5 mg/dL [18]. Acute renal failure was defined as a serum creatinine level of >1.3 mg/dL [19]. Acute respiratory failure was defined as a condition of respiratory failure requiring intubation and mechanical ventilation for more than 24 hours, regardless of the fraction of inspired oxygen [20]. Coma was defined as a Glasgow Coma Scale score of 3 to 8 [21].

### Statistical analysis

Continuous variables are expressed as means and standard deviations and categorical variables as numbers with percentages in brackets. All data were tested for normality of distribution and equality of standard deviations prior to analysis. For comparisons between patient groups, we used the Student’s t test for quantitative variables and Chi-square or Fisher’s exact tests for categorical variables. Mortality data were compared using the Kaplan-Meier method and significance was tested using a log-rank test. An initial univariate Cox regression analysis was performed to compare the frequency of possible risk factors associated with mortality. To control for possible confounding factors, a multivariate Cox regression analysis (stepwise backward approach) was performed with the factors that were significant in univariate models (P<0.05) and met the assumptions of a proportional hazard model. P<0.05 was considered to be statistically significant. All analyses were performed using SPSS Version 20.

## Results

Table 1 presents the demographic characteristics of the patients with CO poisoning, stratified according to status at discharge, namely alive (survivor, n = 242) or dead (non-survivor, n = 19). Most of the patients were middle-aged adults (33±19 years), and were referred to our hospital in a relatively short period of time (6±10 hours). The mean blood carboxyhemoglobin level was 21.9±17.6%. Approximately half of the cases (49.4%) attempted suicide by charcoal burning. Nevertheless, there were no significant differences in baseline variables between survivors and non-survivors.

**Table 1:** Baseline characteristics of patients with CO poisoning, stratified according to status at discharge as alive (survivors) or dead (non-survivors).

Variable	Total (n = 261)	Survivor (n = 242)	Non-survivor (n = 19)	P value
Age, year	33±19	32±19	35±17	0.578
Male, n (%)	142 (54.4)	135 (55.8)	7 (36.8)	0.110
Carboxyhemoglobin, %	21.9±17.6	21.3±16.9	29.4±25.1	0.068
Time elapsed between poisoning and hospital arrival, hour	6±10	6±10	4±3	0.415
Intentional by burning charcoal, n (%)	129 (49.4)	120 (49.6)	9 (47.4)	0.852
Previous suicide attempt	48 (18.4)	45 (18.6)	3 (15.8)	0.916
Hypertension, n (%)	15 (5.7)	14 (5.8)	1 (5.3)	0.925
Diabetes Mellitus, n (%)	7 (2.7)	6 (2.5)	1 (5.3)	0.470
Coronary artery disease, n (%)	11 (4.2)	10 (4.1)	1 (5.3)	0.813
Pulmonary disease, n (%)	9 (3.4)	8 (3.3)	1 (5.3)	0.653
Renal disease, n (%)	8 (3.1)	8 (3.3)	0 (0)	0.421
Neurological disease, n (%)	8 (3.1)	8 (3.3)	0 (0)	0.421
Alcohol consumption, n (%)	65 (24.9)	61 (25.2)	4 (21.1)	0.680
Smoking habit, n (%)	85 (32.6)	80 (33.1)	5 (26.3)	0.722

The CO was extremely dangerous and produced many serious complications after exposure (Table 2). The complications included fever (26.1%), hypothermia (9.6%), respiratory failure (34.1%), shock (8.4%), myocardial infarction (8.0%), gastrointestinal upset (34.9%), hepatitis (18.4%), renal failure (25.3%), coma (18.0%), and rhabdomyolysis (21.8%). Furthermore, it was noted that non-survivors suffered greater incidences of hypothermia (P<0.001), respiratory failure (P<0.001), shock (P<0.001), hepatitis ((P = 0.016), renal failure (P = 0.003), and coma (P<0.001) than survivors.

**Table 2:** Clinical manifestations of patients with CO poisoning, stratified according to status at discharge as alive (survivors) or dead (non-survivors).

Variable	Total (n = 261)	Survivor (n = 242)	Non-survivor (n = 19)	P value
Fever, n (%)	68 (26.1)	63 (26.0)	5 (26.3)	0.961
Hypothermia, n (%)	25 (9.6)	13 (5.4)	12 (63.2)	<0.001***
Acute respiratory failure, n (%)	89 (34.1)	70 (28.9)	19 (100.0)	<0.001***
Shock, n (%)	22 (8.4)	7 (2.9)	15 (78.9)	<0.001***
Acute myocardial infarction, n (%)	21 (8.0)	17 (7.0)	4 (21.1)	0.088
Acute trointestinal upset, n (%)	91 (34.9)	81 (33.5)	10 (52.6)	0.226
Acute hepatitis, n (%)	48 (18.4)	40 (16.5)	8 (42.1)	0.016*
Acute Renal Failure, n (%)	66 (25.3)	55 (22.7)	11 (57.9)	0.003**
Coma, n (%)	47 (18.0)	31 (12.8)	16 (84.2)	<0.001***
Acute rhabdomyolysis, n (%)	57 (21.8)	55 (22.7)	2 (10.5)	0.397

Note:

*P<0.05,

**P<0.01,

***P<0.001

As shown in Table 3, laboratory examinations confirmed that non-survivors had poorer arterial blood gas profile, as well as renal and liver functions, than survivors. However, the incidence of symmetrical bilateral globus pallidus necrosis, a delayed feature of CO toxicity, did not differ between survivors and non-survivors (P = 0.185).

**Table 3:** Laboratory findings of patients with CO poisoning, stratified according to status at discharge as alive (survivors) or dead (non-survivors).

Variable	Total (n = 261)	Survivor (n = 242)	Non-survivor (n = 19)	P value
Blood tests:				
pH	7.354±0.140	7.376±0.091	7.090±0.291	<0.001*
PCO_2,_ mmHg	35.9±10.5	35.2±8.8	45.1±20.5	<0.001***
PO_2_, mmHg	173.7±138.5	175.4±137.0	152.7±159.1	0.517
HCO_3,_ mmol/L	19.8±5.2	20.3±4.6	13.7±7.3	<0.001***
Base deficit, mEq/L	-4.961±6.748	-4.006±5.195	-15.924±11.591	<0.001***
SaO_2_, %	92.6±14.5	93.3±13.1	84.6±25.6	0.018*
White blood cell, 1000/uL	21.3±9.6	21.6±10.0	18.3±11.9	0.885
Platelets, 1000/ul	247.8±100.8	249.6±97.6	227.3±13.3	0.357
Hemoglobin, g/dl	13.8±2.6	13.9±2.5	12.7±3.3	0.048*
Blood urea nitrogen, mg/dl	21.9±26.3	20.1±23.7	40.8±40.8	0.002**
Creatinine, mg/dl	1.6±2.1	1.5±2.0	3.4±3.1	<0.001***
Creatine kinase (MB)	61.9±162.3	61.8±169.2	62.7±65.2	0.987
Creatine kinase (total)	15418.3±39631.2	14850.6±39378.5	33962.7±52846.6	0.413
Myoglobin, ng/ml	31627.7±13003.5	31223.6±134328.0	36073.0±74257.9	0.931
Troponin I, ng/ml	3.3±5.8	3.1±5.4	8.0±11.0	0.061
Calcium, mg/dl	8.2±1.1	8.2±1.1	7.9±1.3	0.423
Inorganic phosphate, mg/dl	3.7±1.7	3.5±1.4	4.6±3.1	0.048*
Potassium, mEq/l	4.1±1.0	4.1±0.7	4.2±2.5	0.627
Aspartate aminotransferase, U/L	169.0±517.3	139.2±354.4	591.0±1499.9	0.002**
Alanine aminotransferase, U/L	111.8±269.7	94.0±228.2	518.7±664.7	<0.001***
Alkaline phosphatase, U/L	62.5±35.0	57.8±26.8	151.7±59.0	<0.001***
Total bilirubin, mg/dl	0.9±0.6	0.8±0.5	1.8±1.4	0.002**
Albumin, g/dl	3.4±1.1	3.5±1.1	2.8±1.1	0.104
Radiographic studies:				
Globus pallidus necrosis, n (%)	44 (16.9)	41 (16.9)	3 (15.8)	0.185

Note:

*P<0.05,

**P<0.01,

***P<0.001

At our hospital, all CO patients were treated with a high concentration of oxygen therapy using a non-rebreather mask (Table 4). Hyperbaric oxygen therapy was used in 18.8% of the patients. In addition, all deaths were from the shock group and all patients resuscitated from cardiac arrest died in the hospital. None of the survivors suffered from cardiac arrest.

**Table 4:** Treatments of patients with CO poisoning, stratified according to status at discharge as alive (survivors) or dead (non-survivors).

Variable	Total (n = 261)	Survivor (n = 242)	Non-survivor (n = 19)	P value
Oxygen therapy via non-rebreather mask, n (%)	261 (100.0)	242 (100.0)	19 (100.0)	1.000
Hyperbaric oxygen therapy, n (%)	49 (18.8)	48 (19.8)	1 (5.3)	0.249
Cardiac arrest, n (%)	19 (7.3)	0 (0)	19 (100.0)	<0.001***
Follow up duration, month	15.0±32.1	16.3±33.2	0.5±0.8	0.039*

Note:

*P<0.05,

***P<0.001

**Fig 1:**
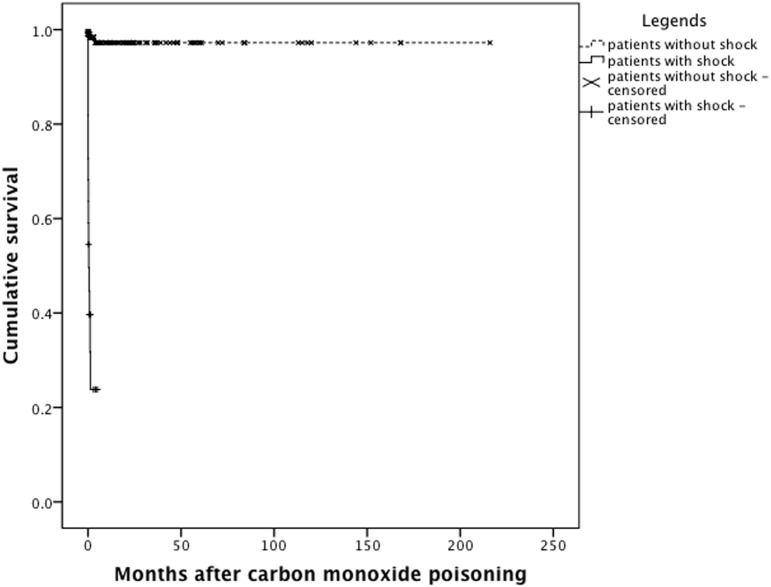
Kaplan-Meier analysis. It was found that patients with shock suffered greater cumulative mortality than patients without shock (Log-rank test, Chi-square 147.404, P<0.001).

Using a multivariate Cox regression model (Table 5), it was determined that shock status was a significant predictor of mortality after CO poisoning (OR 8.696, 95% CI 2.053–37.370, P = 0.003). In other words, a CO poisoned patient with shock was nearly 9 times more likely to die than a patient without shock. Finally, Kaplan-Meier analysis confirmed that patients with shock suffered greater cumulative mortality than without shock (Fig. 1, Log-rank test, Chi-square 147.404, P<0.001).

**Table 5:** Analysis of mortality for patients with CO poisoning (n = 261).

	Univariate Cox regression analysis			Multivariate Cox regression analysis		
	OR	95% CI	P value	OR	95% CI	P value
Arterial ph	250.000	45.455–1000.000	<0.001***	4.785	0.707–32.258	0.109
Hypothermia	19.231	7.463–50.000	<0.001***	1.883	0.586–6.061	0.288
Respiratory failure	142.857	2.532->1000.000	0.016*	>1000.000	0.483->1000.000	0.904
Shock	52.632	17.241–166.667	<0.001***	8.696	2.053–37.370	0.003**
Acute hepatitis	2.976	1.196–7.407	0.019*	1.389	0.497–3.876	0.532
Acute Renal Failure	3.831	1.543–9.524	0.004**	1.017	0.355–2.914	0.975
Coma	23.256	6.803–76.923	P<0.001***	2.564	0.488–13.514	0.266

Note:

*P<0.05,

**P<0.01,

***P<0.001, OR odds ratio, CI confidence interval

## Discussion

The mortality rate for patients suffering from CO poisoning at our center was 7.3%. In a retrospective review of the medical records of 1505 patients at Virginia Mason Medical Center in Seattle from 1978 to 2005 [22], the short-term mortality rate was reported to be 2.6%. After reviewing previous publications (i.e., 2359 patients in total) in literature, this group also reported a short-term mortality rate of 3.9% [22]. Furthermore, according to another retrospective study from the same institution [23], it was revealed that a total of 162 subjects had died in 11741 person-years. The expected number of deaths was 87 (standardized mortality ratio 1.9; 95% CI 1.6–2.2). Therefore, it was unclear whether the difference in mortality rates between our center and other center was due to different baseline characteristics. For example, approximately half of our cases (49.4%) attempted suicide by burning charcoal, but this method of suicide was unusual in the studies by Hampson et al [22, 23].

In the present study, there was no significant difference in baseline blood carboxyhemoglobin levels found between survivors and non-survivors (P = 0.068; Table 1). Furthermore, carboxyhemoglobin level was not identified as a risk factor for mortality (Table 5). Theoretically, the using the percentage of carboxyhemoglobin as a measure of CO poisoning severity or for predicting outcome is limited because carboxyhemoglobin levels are influenced by removal from the source of CO and any oxygen treatment given prior to the measurement of carboxyhemoglobin levels. In a study performed by Hampson and Hauff [22], it was revealed that severe metabolic acidosis (P<0.001) and the need for endotracheal intubation (P = 0.002) were the factors most strongly associated with mortality. Similarly, carboxyhemoglobin was not determined to be a significant risk factor for mortality (P>0.05) [22]. Additionally, people with co-morbidities that make them more sensitive to the hypoxia associated with CO can present with symptoms of poisoning at carboxyhemoglobin levels that are low or within the normal range [24].

Conversely, shock was found to be a significant risk factor for mortality after CO poisoning (Table 5). Our data also showed that non-survivors had a higher incidence of shock than survivors (Table 2). The mechanism of CO toxicity resides in the ability of CO to bind to hemoglobin molecules with high affinity, displacing oxygen and generating CO, which is virtually ineffective at delivering oxygen to the tissues. The organs with the highest demand for oxygen, such as brain and heart, are more vulnerable to injury [25], and could result in shock after severe cardiac damage. Although the etiologies of shock might be multifactorial in cases of CO poisoning, the shock variable predicted mortality after CO poisoning in our population.

There were many limitations regarding the findings of this study due to it being retrospective in nature, small sample size, lack of standard indications for hyperbaric oxygen therapy, lack of magnetic resonance neuroimaging studies [26, 27], lack of plasma inflammatory biomarkers analysis [28], and short follow-up duration. For example, Hox et al [29] revealed that diffusion tensor imaging was a valuable tool for assessing the severity of brain injury and a predictor of outcome in patients with delayed encephalopathy after CO poisoning. Thom et al [28] also demonstrated a complex pattern of elevations in acute phase reactants and proteins associated with inflammatory responses including chemokines/cytokines and interleukins, growth factors, hormones, and an array of autoantibodies of the plasma samples of patients with CO poisoning. Thus, more researches in this area are warranted.

In summary, the mortality rate for patients with CO poisoning at our center was 7.3%. Furthermore, it was concluded that shock status was a significant predictor of mortality after CO poisoning (P = 0.003), and a CO poisoned patient with shock was nearly 9 times more likely to die than a patient without shock. Therefore, early recognition and aggressive therapy of shock, by means of abundant fluid resuscitation, use of catecholamines and other adjuvant drugs, are of pivotal importance to improve the outcome of these patients.
